# Introducing *Communications Medicine*

**DOI:** 10.1038/s43856-021-00009-z

**Published:** 2021-06-30

## Abstract

*Communications Medicine* is publishing its first articles today. We are an inclusive and open access medical journal that aims to facilitate and disseminate discovery that will promote health for all and improve the lives of those experiencing or living with disease.

The COVID-19 pandemic has brought to the fore how powerful collaboration can be, when all stakeholders involved in translational and clinical research, public health and healthcare, are focused on a common goal. However, it has also highlighted the many inequalities in clinical outcomes, access to healthcare, and inclusive representation in medical research. These two learning points are by no means exclusive to COVID-19, and *Communications Medicine* believes there is a role for journals in promoting cross-field collaboration, addressing health inequalities, and nurturing a more diverse representation in medical research.

*Communications Medicine* believes there is a role for journals in promoting cross-field collaboration, addressing health inequalities, and nurturing a more diverse representation in medical research.

*Communications Medicine* is an open access, broad scope journal publishing high-quality research of interest to a specialist audience, across all medical specialties and public health fields, as well as research about translating biomedical discovery to clinic practice or to improve population health. We are equally interested in well-established fields as we are in new ones, and our scope will undoubtedly continue to evolve to capture emerging areas. As illustrated by some of the articles we are publishing today, we also welcome work at the intersection with other disciplines, such as computational science, physics or engineering that will be of interest to a medical audience. Our multidisciplinary scope is an opportunity to foster collaboration across fields and potentially help generate new ideas at the intersection of the communities we serve. We will aim to encourage such collaborations and discussions through a range of commentary article types.

We also want to be a platform for all topics and participants in medical research and healthcare. We will foster discussion on health inequalities and diverse representation in medical research and healthcare. We will also champion patient-centric medical research and healthcare by having the voices of those experiencing or living with disease on our pages. To facilitate public and patient engagement with medical research we have introduced a Plain Language Summary in our Article format that should be accessible to anyone without a medical or scientific background. We hope that this summary, combined with our open access model, will help to make medical information accessible to everyone and facilitate a more involved and empowered role for the public and patients in health-related decisions.

We are also making the data behind our content publicly available and our process transparent, to help build trust in medical research. We encourage authors to make supporting materials, datasets and code available to readers, and all articles must describe in detail how these can be accessed (within legal and ethical constraints). We also offer transparent peer review, giving authors the choice to have the peer reviewer reports published alongside their manuscripts providing insight into the reviewing process.

We have a unique editorial model in which an in-house team of scientific editors with in-depth research experience and an Editorial Board of active researchers and/or clinicians collaborate in selecting and developing manuscripts through constructive and collegial peer review. External academic editors also take responsibility for individual manuscripts while maintaining close communication with in-house editors regarding all decisions. This allows us to provide authors with excellent service while also keeping the real-world researcher and medical practitioner experience at the forefront of our decision-making.

We are excited to publish our first articles today, covering diverse topics within our scope. Unsurprisingly, three articles focus on different aspects of COVID-19 epidemiology; serology of a unique outbreak with a superspreader event (Knabl et al.), the impact of different non-pharmaceutical interventions on virus dynamics (Pereda-Loth et al.), and sex-specific risk factors associated with poor COVID-19 outcomes (Jun et al.). Two other studies illustrate how computational tools can assist in medicine, by predicting prostate cancer-specific mortality better than pathologists (Wulczyn et al.) or investigating the mechanical safety of a personalised high tibial osteotomy (MacLeod et al.).

Reflecting the journal’s mission to provide a platform for commentary and discussion, we are also publishing two Comments today. Shinjini Kundu discusses how artificial intelligence might change medical training, and Kurtzhals and colleagues mark the insulin discovery centenary by reflecting on the role of this protein in shaping protein-based therapies for chronic disease and how it will continue to do so.

We have two editorial picks this month in Research Highlights. A phase II clinical trial by Mintun and colleagues published in *The New England Journal of Medicine* reports tentatively encouraging results of targeting aggregated amyloid-β with a monoclonal antibody for the treatment of early Alzheimer’s disease^1^. A translational study published in *Nature* by Musunuru and colleagues demonstrates the safety and feasibility of targeting *PCSK9* in vivo using CRISPR base editing in non-human primates to reduce LDL cholesterol, opening up a potential avenue to tackle hypercholesterolemia in humans^2^.

We would like to thank our first authors, reviewers, Editorial Board members, and readers—you have turned *Communications Medicine* into a reality and we are grateful for your trust. We are only getting started and it takes a village to grow a journal. We look forward to welcoming you in ours!
